# Fabrication of slag/CKD one-mix geopolymer cement reinforced by low-cost nano-particles, mechanical behavior and durability performance

**DOI:** 10.1038/s41598-024-53023-1

**Published:** 2024-01-31

**Authors:** Fayza S. Hashem, Taha A. Razek, Hani A. Mashout, Faten A.Selim

**Affiliations:** 1 Chemistry Department, Faculty of Science, Ain Shams University, Cairo, Egypt; 2 Faculty for Environmental Studies and Research, Ain Shams University, Cairo, Egypt

**Keywords:** Environmental sciences, Chemistry, Engineering, Materials science

## Abstract

CKD is a byproduct of the cement industry, and its accumulation in the surrounding represents one of many issues associated with this industry. In this study, CKD was utilized in the fabrication of one-mix geopolymer cement (GP) composite as an economical and environmental solution for disposal of this byproduct. The mechanical properties and durability behavior during various deterioration actions were inspected. The obtained findings demonstrated that, replacing slag by CKD in the fabricated GP could cause an elongation in the setting times and reduction in the compression strength of approximately 50%. However, GPs containing CKD offered an accepted resistance to irradiation by γ-rays and to firing action. Reinforcing the GPs with nano Fe_3_O_4_ (NF) or nano TiO_2_ (NT) accelerated the geopolymerization reaction and offered mechanical properties surprising the control mix, this was related to the micro-filling and catalytic actions of the NPs which supported the formation of symmetrical and organized clusters of CSHs and CASH gel as shown in SEM micrographs. The reinforcing mixes surpassing the control mix in the protection against intrusion of sulfate ions which they could retain about 92% of their strength after 4 months of exposure while the control mix retained 80%. Furthermore, they showed a superior resistance to the destructive effect of irradiation by high dose gamma rays up to 1500 *kGy* and they retained ~ 75% of their strength after irradiation while the control mix was kept at only 35%. The fabricated composites are recommended for usage in many applied construction fields.

## Introduction

Cement kiln dust (CKD), a fine powdered material, is one of the byproducts collected from exhaust gases emitted during PC production. According to Portland cement association (PCA) survey in 2006, approximately 16% of clinkers were diagnosed with CKD^1–3^. The main components of CKD are unreacted raw feed, partially calcined raw feed, CaO particles and fuel ash^4,5^. Minor amounts of alumina and metal oxides are also present in CKD. CKD possess alkaline pH due to its high content of alkali metals such as Na, K and Ca. The deposition of CKD in the surrounding environment may cause serious health problems for all living organisms and human being as well. For further calcination. Sometimes to be used as landfill or recycled to the cement kiln for further calcination. Sometimes and due to high alkalinity of CKD, it is difficult to recycle CKD inside the cement kiln^6^. Many research attempts were carried out to benefit the usage of CKD including blending with OPC^7,8^ or as a waste stabilizer^9,10^. Recently, CKD was used in the production of building materials as an alkaline activator for many pazzolana materials^11,12^.

However, dust emissions are not the only problems associated with PC manufacture. Natural resource conservation and high energy demand are challenges associated with this industry^13^. More emphasis is being devoted to alkali-activated cement or geopolymer composites (GPs) as superior binders that can replace PCs in their application. Furthermore, geopolymer binders (GPs) are composites that are free of PC and their fabrication do not depend on the firing of limestone, which reduces CO_2_ emission in the surrounding. Geopolymers (GPs) can be chemically prepared from natural clays, natural fibers or from industrial solid waste precursors. Metakaolin^14,15^ and basalt fibers^16,17^ are examples of natural resources that have been used in GP fabrication, while, blast furnace slag^18^, red clay brick waste^19,20^, kiln dust^21^, concrete wastes^22^ and marble dust^23^ are examples of solid wastes that have been incorporated as GP percoursers. From a practical point of view, GPs have accepted performance similar to PCs in some of their applications, and they also offer additional advantages, such as diminished setting times, rapid strength development^24,25^. Additionally, GP offers better endurance in both acidic and corrosive ionic environments^26,27^ and in many cases, GP binders outperformed OPC in firing actions^28^. However, the properties of GPs are strongly affected by many factors, such as; the composition of the starting precursors, the applied alkaline activators, the GP concentration and, ultimately, the curing or polymerization conditions^15,29–31^. However, there are many dangerous problems that limit the expanded use of GP, which can be associated with handling concentrated solutions of alkali activators. Recently, ready-mix geopolymer materials were developed as examples of GP containing a dry mixture of aluminosilicate precursors and a dry activator as one mix^19,32^. In the ready mix, water was directly mixed with the other concrete components in a way like traditional cement which ease and spread its usage^33–35^. However, to the best of the authors’ knowledge, studies concerning with the durability performance of one-part geopolymers, especially those containing byproducts as CKD, for various deterioration actions are limited which calls for more research. Hence, the current study is an effort to develop the mix design of GP composites, containing CKD approaching the optimizes GP's hardened and fresh state properties.

The reinforcement of cementitious materials or concrete by nanoparticles is a new technique that can be used to modify granular matrices. Nano-titania (NT), nano-magnetite (NF), nan-osilica (NS), carbon fibers (CFs) nano clays (NCs), and nano calcium carbonate are examples of these materials that have microscopic reinforcement actions^36–41^. Recently, extensive studies have been performed to examine the effect of introducing nanoparticles inside a GP matrix^42,43^. In the present work, the performance and mechanical properties of slag/CKD -based one-part geopolymers were examined. A mixture of Na_2_Si_2_O_3_ and NaOH was used as an alkaline activator for the geopolymerization process. Nano-Fe_3_O_4_ and nano-TiO_2_ were incorporated inside the prepared GP matrix as examples of inexpensive and easily prepared nanoparticles. The performance of the developed GP in fire tests up to 750 °C, during sulfuric acid exposure and during SO_4_^–2^ ion penetration was monitored. Additionally, the impact of a high dose of gamma radiation on the developed hydrates was assessed.

## Practical routine

### Materials resources and characterization

**Figure 1:**
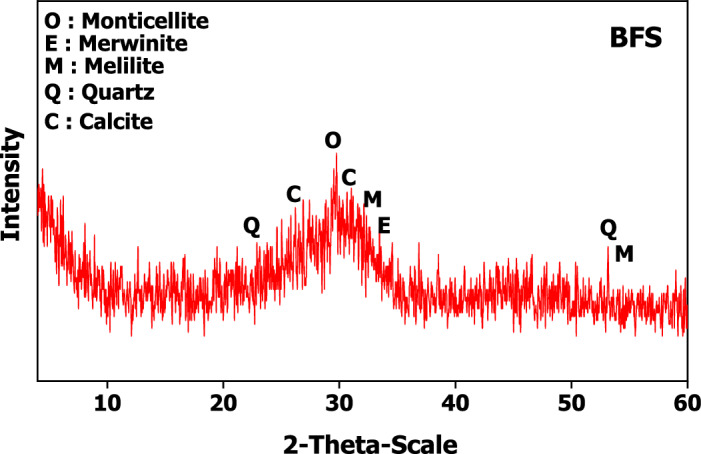
XRD of BFS.

**Figure 2:**
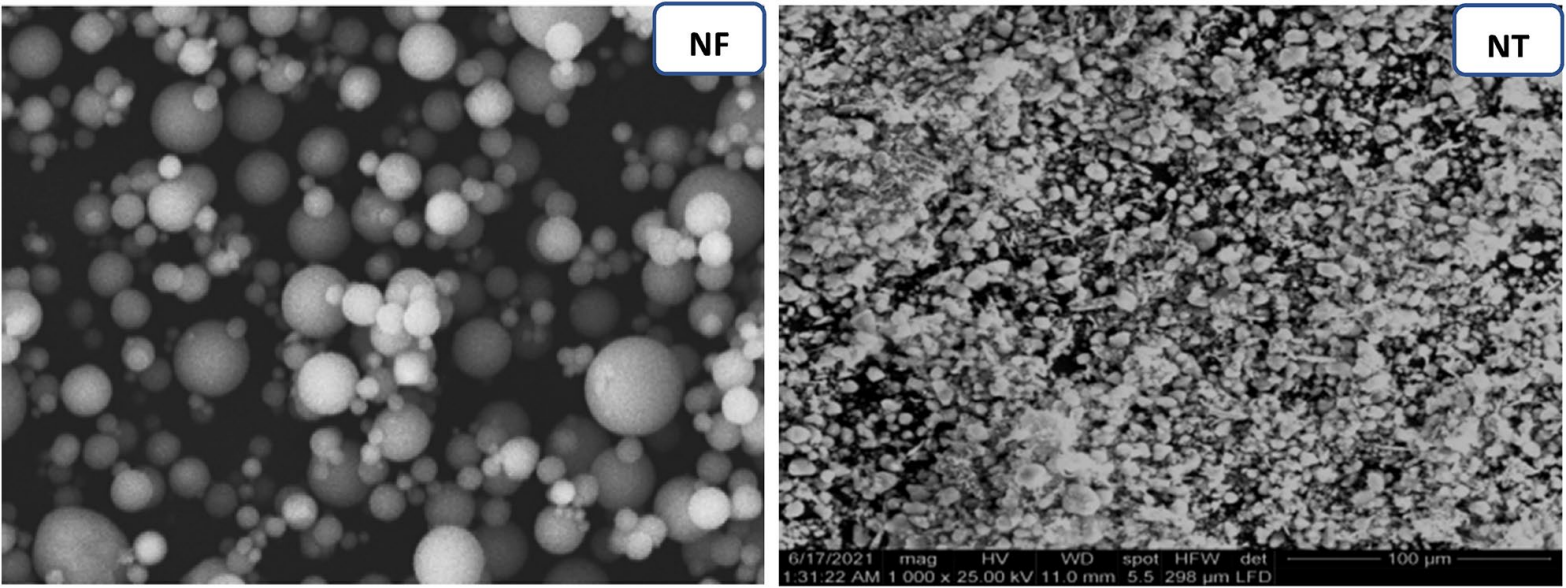
SEM of NF and NT.

The raw precursors used in this study were:

- Blast-furnace slag (BFS) was sourced from iron and steel factory, Helwan governorate, Egypt. BFS has Blaine specific surface area of 2883 cm^2^/g.
- Cement kiln dust (CKD) which was collected from cement kiln, Lafarge Egypt cement Company (Suez, Egypt). Suez cement Company. CKD is sieved to particles sized ≤ 125 µm of Blaine surface area 5690 cm^2^/g. The mineralogical oxide (%) of BFS and CKD is assessed by XRF (XRF, model PW-1400, Xios) and represented in Table 1. XRD diffractometry of BFS (assessed by XRD, model Xpert-2000, Philips) was illustrated in Fig. 1.
- Sodium silicate and sodium hydroxide were used as alkaline activators. They were supplied from Al-Salam Association, 6 October District, Egypt. Both sodium silicate and sodium hydroxide were added to the dry mix of GP by 15% (5% NaOH + 10% Na_2_Si_2_O_3_) to form a one-part mix^44^. Nano magnetite Fe_3_O_4_ (area = 86.5 m^2^/g, d = 0.045 g/ml) prepared by sol gel method as mentioned in our pervious study^45^. Nano TiO_2_ (purity ≥ 99%, d = 0.04–0.06 g/ml, Area = 101.6 m^2^/g) is supplied from Al-Salam Association, Egypt. Figure 2 shows SEM micrographs of NT and NF. According to SEM micrographs for NF showed separated spherical particles with size ranged between 2 and 5 µ while NT appeared as tiny particles with size less than 100 nm.

**Table 1:** Chemical oxide (%) composition of the raw materials**.**

Oxide (%)	BFS	CKD
Al_2_O_3_	7.11	15.34
CaO	35.38	2.71
SiO_2_	38.33	62.4
Fe_2_O_3_	2.96	3.23
MgO	5.40	0.8
K_2_O	0.76	1.43
Na_2_O	0.28	1.83
SO_3_	1.29	0.04
Cl^−^	0.01	0.03
L.O.I	9.49	10.7

### GP mix design

In this research GP was fabricated using BFS and CKD. BFS/CKD based GP was prepared by replacing 0, 20, 40, 60 wt% of BFS by CKD. The solid alkaline activator was introduced to the dry mix by 15% of the total mass of the mix (10% Na_2_Si_2_O_3_, 5% NaOH). Each dry mix (including the dry raw precursors and the solid activator) was agitated for 6 h in a porcelain ball mill to ensure the homogeneity of the mix. Table 2 demonstrates the notation and design of each dry mix. The prepared GP was reinforced by 0.5 and 1% nano magnetic Fe_3_O_4_ or nano TiO_2_. The nanoparticles were first sonicated for 1 h with the mixing water in presence of 3% super plasticizer (water reducer) using ultrasonic homogenizer (LUHS0A12, 650W, 220 V/50HZ). Each solid dry mixes were mixed with water for 3 min to form a homogenous paste. Then each paste was shaped into cubic samples using cubic molds (2.5*2.5*2.5 cm^3^) and kept at *RH* ≈ 98 ± 2% for 24 h to attain the final setting. After 24 h, the solidified cubic samples were kept in humid chambers with relative humidity (≥ 99%) at temperature 25 °C to continue the geopolymerization reactions^14,24^.

**Table 2:** Mixes notation and their composition.

Mix	Composition (wt%)	Water of consistency (ml)
B	100% BFS	120
CKD20	80%BFS + 20% CKD	125
CKD40	60%BFS + 40% CKD	138
CKD60	40%BFS + 60% CKD	150
CKD20-T1	80%BFS + 20% CKD + 0.5% NT	123
CKD20-T2	80%BFS + 20% CKD + 1% NT	128
CKD20-F1	80%BFS + 20% CKD + 0.5% NF	122
CKD20-F2	80%BFS + 20% CKD + 1% NF	125

### Testing

The fabricated BFS/CKD based geopolymer was subjected to the following tests:

- Setting times: According to ASTM C191, the Vicat apparatus was used to measure the initial and final setting times for all the freshly developed geopolymer pastes^46^. This apparatus comprises a frame that contains a mobile rod, equipped with a disc at one side and a needle that can be affixed at the opposite end.
- Compression strength tests were done using a Ton Industric machine (West Germany) for maximum load of 60 tons. The test was performed on three cubic specimens, that represent each geopolymer mix, after 3, 14 and 28 and 90 days according to ASTM C109 M16-a^10,47^. The compressive strength test was performed on three cubes and the mean value was adopted with an error range ± 5%. Water of consistency were measured for each mix according to ASTM C187^48^.

### Durability tests

The prepared geopolymer cements were assessed to four different durability circumferences. The tests were performed on 28-day-old drying samples, and the testes were carried out on individual sets represented each mix as follow:

#### Firing test

A firing resistance test was performed on 28-day-old drying samples by exposing the samples to three firing temperatures, 250, 500 and 750 °C, for three hrs. After firing, the fired samples were gradually cooled in desiccators to room temperature. The compressive strength test was subsequently performed on three heated cubes, as described in the compressive strength section, and the Residual strength (RS)t was assessed via Eq. (1):

1 $$({\text{RS}})\mathrm{t }= \frac{(C.S )t}{(C.S.)0} x 100$$

(C.S.)_t_: compressive strength after firing.

(C.S.)_0_: compressive strength before firing (28-days hydration).

#### Acid attack

Attack by H_2_SO_4_ acid (2N) was done by immersing the hydrated 28-days samples in the acid solution for 2 months and the solutions were renewed weekly^49,50^. After 15, 30 days and 2 months of curing, samples were removed and assessed to compressive strength test.

#### Penetration of SO_4_^2−^ ions

Attack by sulfate ions is tested by immersing the hydrated 28-days samples in MgSO_4_ solution (7%) for 4 months.

#### Irradiation by γ-ray

Radiation resistance was examined via exposure the dried 28 days sample to gamma ray source (Co^60^, dose rate 985 kg/h) to 500, 1000 and 1500 kGy with a dosing rate 1.4 kGy/h. After irradiating the sample, the compressive strength test was assigned and the residual strengths (RS %)_rad_ were calculated using Eq. (2):

2 $$({\text{RS}})\mathrm{rad }= \frac{(CS)rad}{\left(CS\right)0}\times 100$$

### Phase composition and microstructure

GP phases that developed during the hydration of the various geopolymer specimens were identified using XRD. XRD was performed using cobalt target (λ = 0.17889 nm), and filter made of nickel under 40 kV and 40 mA with a scanning range spanned from 5 to 60 (2θ°), and a scanning speed of 1s/step and a resolution of 0.02/step. The microstructure of various GP samples was explored using Scanning electron microscope test (SEM-Quanta 250 FEG equipment).

## Results and discussion

### Setting times and water of consistency

**Figure 3:**
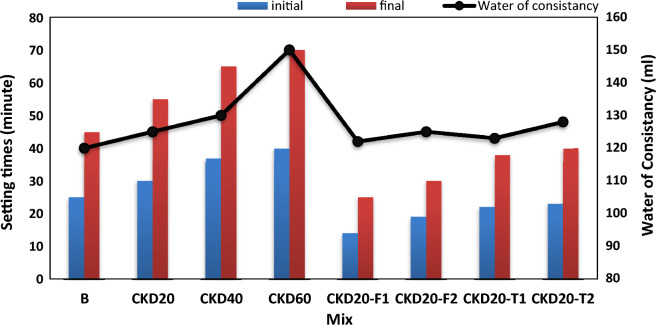
Setting times and water of consistency for various geopolymer mixes.

Setting times are crucial factors for deciding the applied use of GP or how it can be handled or shaped. Figure 3 illustrates the initial and final setting times and the standard water of consistency of BFS/CKD based geopolymer. Geopolymer based on BFS only (B) offers fast setting times, 25 and 45 min for initial and final setting times respectively. The fast-setting times recorded for ready mix GP can be attributed to the heat liberated from the exothermic dissolution of solid NaOH placed in the mix as alkaline activator^24^. Blending slag by CKD causes a delay in setting times and they became longer as the % of CKD replacement increases in the GP mix. *GP* mix contains 60% CKD (mix CKD60), records 40 and 70 min for initial and final setting times respectively. Besides, the blended mixes show a higher water demand than the control mix. Such finding can be attributed to change in the composition of the GP mix by blending. According to literature, calcium-rich aluminosilicate precursor (BFS) would induce a rapid setting and high early strength^51^ and replacement of slag by high calcium oxide precursor (as CKD) retard the workability in a one-part geopolymer^52^. Besides, increasing the water demand of GP mix reduces the alkalinity of system which favor the formation of Al-rich gel (has a low binding properties) at the early stages of geopolymerization and so delay the setting process. Also, using high Water/Solid ratio boosts the distance between the inter-particles or a decrease of the ‘‘particle–particle’’ interaction^53–55^.

Admixing blended GP mixes by 0.5 or 1% nano particles (NF or NT) causes a great diminish in the setting times to be values comparable to those obtained in case of control (B mix). NF offers fast setting than NT. According to Hajimohammadi et al., dispersion of nano particles in the one-part geopolymer matrix performs a seeded nucleation phenomena^38,56–59^. This nucleation hinders the absorption of Al on silica particles which facilitates their dissolution and release inside the matrix leading to build up GP chains quickly.

### Compression test

**Figure 4:**
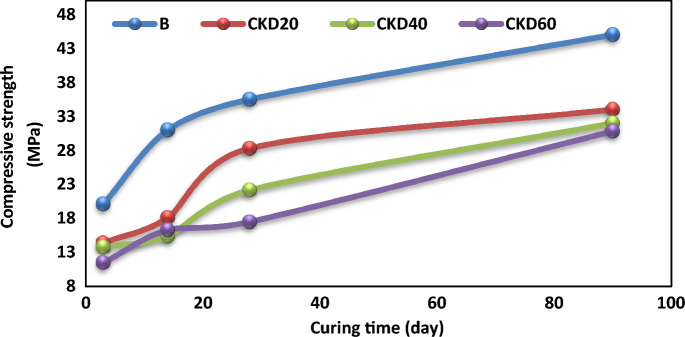
Compressive strength versus curing times for various geopolymer mixes.

The variation of the compression strength (MPa) with the geopolymerization time is illustrated in Fig. 4. All the BFS/CKD based GP and the control mix (B) showed a continuous boosting in the compressive strength (CS) by aging times up to 90 days. There are two stages of strength development. A rapid stage in which 50–66% of the strength is gained. During this stage, the dissolution of the precursor particles by the action of the alkaline activator mixture starts, leading to the promotion of active species like Ca^2+^; [H_2_SiO_4_]^2−^, [H_3_SiO_4_]^−^ and [Al(OH)_4_]^−^. These species act as monomers that building up the geopolymer network throughout GP matrix^14,60^. In control mix, this rapid stage takes about14 days while in blended mixes, it extended to 28 days which reflects the delay effect occur in blended mixes. The second stage of strength development is a slower and characterized by rapid and continuous development of strength form 28–90 days. All the blended mixes showed low CS than the control. About 50–80% loss in CS for replacing BFS by 20–60% CKD after 28 and 90 days. This loss in CS by increasing with CKD content in GP mix and can be corelated to the increase in the CaO content in expense of Si and Al content. Table 3 demonstrated the oxide and mineralogical composition in various GP mixes as studied by XRF. Obviously, as % of CKD in GP mix increase, Si & Al content decrease with boosting in CaO. This reduces the amount of soluble Si whose promote the geopolymer reaction^14,21,23^. Additionally, soluble Si reorganizes the geopolymer network by building up Si–O–Si and Si–O–Al bonds in GP gels and they also reinforce the connections between particles leading to formation of 3D GP network of poly silalate^61^. According to Hassani and Zhang the concentrations of Ca^2+^ and Na^+^ ions become high when the CKD content increases in fly ash based geopolymer which favor the formation of N–(C)–A–S–H gel with low binding properties^62^.

**Table 3:** Oxide compositions (%) of various geopolymer dry mixes.

Mix	Oxide %							
	SiO_2_	CaO	Al_2_O_3_	Na_2_O	Fe_2_O_3_	MgO	SO_3_	K_2_O
B	40.11	32.38	6.11	9.03	2.16	5.4	1.29	0.76
CKD20	28.11	38.08	4.19	10.03	1.52	4.05	2.35	0.99
CKD40	25.29	38.74	3.38	10.32	1.65	3.15	2.75	1.15
CKD60	22.25	39.36	2.05	14.51	1.59	2.79	2.95	1.19

**Figure 5:**
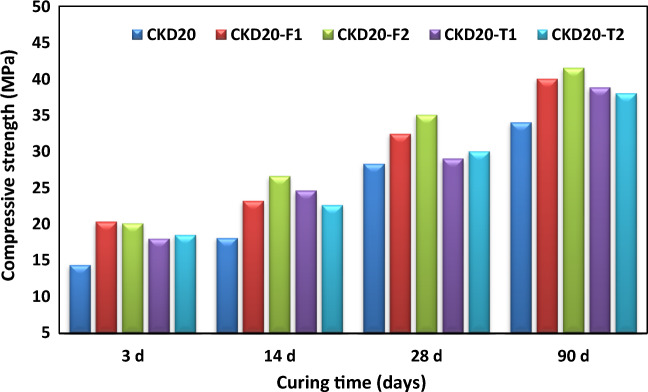
Compressive strength of geopolymer mixes admixed by nano particles.

To improve the low mechanical properties exhibited by slag/CKD geopolymer mixes, CKD20 was admixed by 0.5 or 1% nano particles (NPs). Figure 5 illustrates, the compression strength values for CKD20, CKD20-T1, CKD20-T2, CKD20-F1 and CKD20-F2 on various curing ages. Obviously, the admixed mixes showed an improvement in CS values especially at early curing ages; 3 and 14 days, and for mixes containing NF than those containing NT. Furthermore, the admixed GPs mixes offered CS values may superpass those obtained in control (B) mix. NF offer 43 ~ 22% an improvement in the CS after 3 and 90 days while NT offers 25 ~ 28.5% at the same ages compared to B mix. Such finding is mainly related to the nano-filling effect as well as the nucleation properties of NPs which speed up the geopolymerization process between the initial precursors^63^. Also, NF can react with Ca (OH)_2_ and form a hydrated product called Ilavite which has reasonable hydraulic character^64^. It was demonstrated by Ren et al.^65^ that the NT refines micropores thereby obtaining a larger bonding interface which enhancing the concrete density.

### *Durability test*s

#### *Acid and SO*_*4*_^*−*^* ions attack*

**Figure 6:**
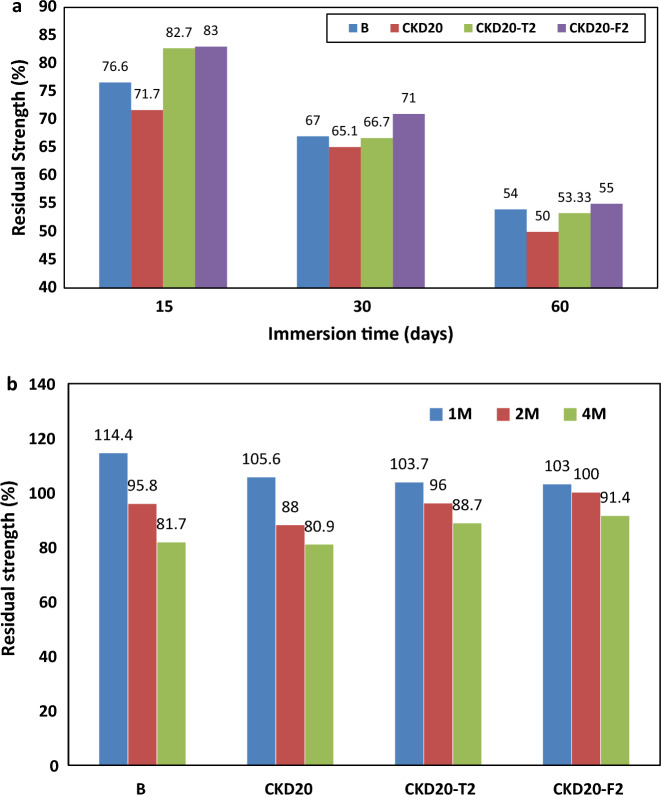
(**a**) Residual strength (%) of geopolymer mixes after immersion in H_2_SO_4_ solution. (**b**) Residual strength (%) of geopolymer mixes after immersion in MgSO_4_ solution.

Figure 6a displays the residual strength (RS%) for B, CKD20, CKD20-T2 and CKD20-F2 after immersing in H_2_SO_4_ solution (2N) up to 60 days. Obviously, all GP suffered from a dramatic deterioration action via their exposure to acid solution as noted by the continuous depletion in CS. About from 50 to 55% loss in CS were recorded for all the studied mixes after 60 days in sulfuric acid. These results are consistent with earlier study on fly ash based geopolymer concrete which reported the loss in CS due to exposure to sulfuric acid solution in the range between 30 and 66%^66,67^. This loss in CS can be related to dissociation of alkali cations by their reaction with acid and the breakdown of alumino-silcates chains in 3-D geopolymer network^63^. Acid exposure also results in the partial removal of aluminum ions in Si–O-Al bonds and formation of Si–OH and Al–OH bonds which terminate the GP chains. This weakness the GP chains and limits their growth.

Figure 6b shows Residual strength (RS%) for various GPs mixtures after immersion in MgSO_4_ solution (7%) up to 4 months. Obviously, the fabricated GP mixes showed a better resistance in MgSO_4_ solution than their behavior in H_2_SO_4_ as indicated by the recorded RS% values compared to those in acid solution. All the studied mixes showed an improvement in the compressive strength during the first month of immersion in MgSO_4_ solution. Such improvement in RS% can be related to the formation and precipitation of gypsum inside the GP pore matrix^48,66,68,69^. This is followed by a gradual loss in RS % during the next period and about ~ 20% loss in strength was recorded for B and CKD20 mixes after 4 months while CKD20-T2 and CKD20-F2 recorded 12 and 9% loss in strength during the same period. This reflecting the extent of reaction of MgSO_4_ with the main binding phases in GP chains (CSH, CASH and NASH)^70^. This interaction reduces the quantity of binding phases in geopolymer matrix and decline the strength^71^. According to Elyamany et al., 20% loss in CS due to immersion of GP in MgSO_4_ solution for 48 weeks and related this loss to the formation of magnesium aluminum silicate hydrate (MASH) gel in the geopolymer matrix^72^. MASH gel has lower mechanical properties than NASH or CASH.

#### Shielding test

**Figure 7:**
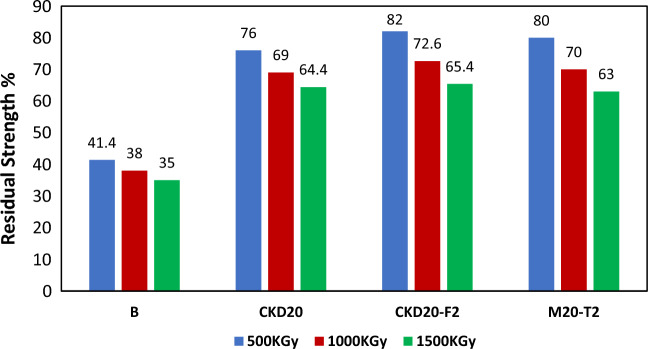
Residual strength (%) of geopolymer mixes after irradiating by γ-ray.

One of the durability testes which can be carried on the fabricated GP is their ability to shield or resist the destructive effect of γ-rays radiation. This test done on 28-day hydrated samples by their exposure to γ-ray of various doses and examine their compression strength after exposure. Figure 7 displays the residual strength (%) of B, CKD20, CKD20-F2 and CKD20-T2 mixes after irradiation using γ-ray source (60Co-γ-cell-220, Atomic Energy Commission, Canada) of various intensities (500, 1000 and 1500 kGy*.* Figure 7 shows that the RS_rad_ decreases with increasing power of the irradiated γ-rays, indicating that the destructive properties of the irradiated rays attack the hydration products^73,74^. The strength of the B mixture is approximately 41.4 ~ 35% after irradiation with 500 or 1500 kGy of γ-rays. However, the blended mixture of CKD20 showed better resistance to the destructive effect of γ-rays than did the control mixture, and approximately 75–65% of the strength was maintained by irradiation at the same doses. This behavior can be attributed to the cross linking of the linear alumino-silicate chains which leads to additional hydration products. In addition, the composite mixture of CKD20-F2 and CKS-T2 also had better γ-ray results. This difference may be due to the filling and catalytic properties of the NPs, which permit the formation of a dense GP microstructure with high resistance to the destructive effects of gamma rays.

#### Firing resistance

**Figure 8:**
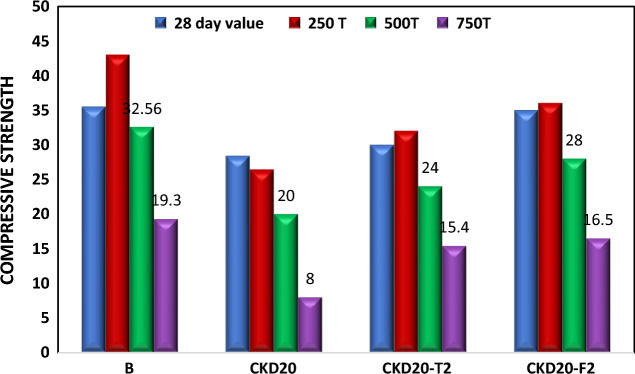
Compressive strength of geopolymer mixes after firing at 250, 500 and 750 °C.

Figure 8 displays the compressive strength of B, CKD20, CKD20-F2 and CKD20-T2 after firing at 250, 500 and 750° C for 3 h and cooled gradually to room temperature then examined for compressive strength test. B mix shows an improvement in CS via heating at 250 °C, then a gradual drop in strength by heating at 500 and 750 °C. CKD20 mix demonstrated a dramatic failure in CS and loss about 30 and 85% of its CS (28-day value) via exposure to 500 and 750 °C respectively. The loss in strength by heating GP at 500 and 750 °C is related to thermal deterioration inside the geopolymer matrix and destruction of the formed hydrates. This deterioration action leads to dehydration and dehydroxylation of the formed hydrates and steam pressure inside GP matrix. This steam induces an internal pressure causes cracks formation and drop in CS. The degree of these actions mainly depends on the types of the formed hydrates which as a sequence, related to the initial precursors from which GP was made. According to literature, GP᾿s calcium rich hydrates had low heat resistance compared to silica or silica/alumina rich hydrates which explain why CKD20 demonstrated a dramatic failure in CS by heating at 500 and 750 °C^21,67,72^. Mixes reinforced by NPs, CKD20-T2 and CKD20-F2 show a slight boosting in CS by heating at 250 °C and they could keep about 80 and 50% from their strength (28-days value) by firing at 500 and 750 °C respectively. Such behavior confirming the role of NPs for increasing the durability of CKD/Slag blended mixes.

### XRD

**Figure 9:**
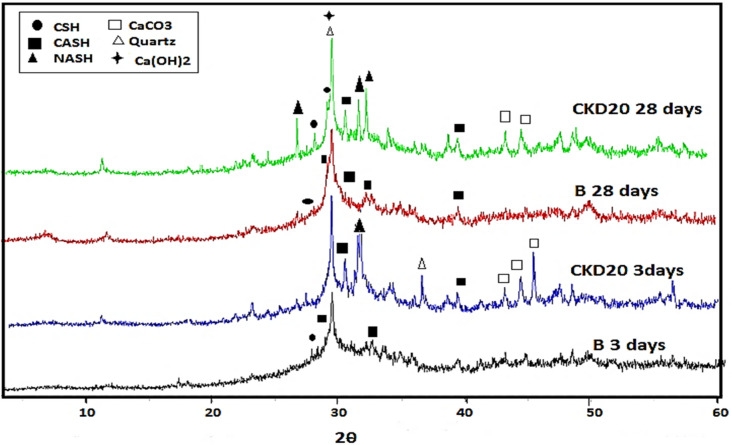
XRD patterns of B & CKD20 mix after 3 and 28 days of curing.

Figure 9 illustrates XRD patterns for mixes B, CKD20 after 3 and 28 days of curing. XRD patterns of B after 3 days displays small peaks appeared at 2θ = 29.54° and 32.21° assigned to CSHs gel and hydrogarnet gel (CASH) respectively as main hydration products of geopolymerization of BFS^21^. CSHs and CASH built the basic skeleton of percolating solids which onset the hardening process^20,68,69^. Peaks of unreacted quartz (2θ = 29.64°) and illite phase are identified in that pattern.

Replacing slag by CKD in GP, CKD20 mix, led to distinctive changes in XRD patterns such as: (i) appearance of peaks assigned to CaCO_3_ (due to interaction of CaO with air), (ii) appearance of highly crystalline peak at 2θ = 32.2 (PDF# 01079-2424) due to garronite (NCASH), and (iii) appearance of weak sharp peak that distinguishes tetragonal Na-alumino-silicate hydrates (NASH) at 2θ = 27.8°, 32.1°. Appearance of these two phases in blended mixes can be attributed to the change in the phase morphology of the polymeric system as a result of exchange slag by CKD. Presence of CKD increases the Ca and Na contents in the system which favours the formation of NCASH gels rather than CSH and CASH gels^63,71^. Besides, NCASH may be formed in the polymeric system due to the transformation of gismondine; a highly calcium-rich phase, to NCASH by exchange process of Ca^2+^ ions by Na^+^ ions in high concentrated alkaline medium^21,75^.

**Figure 10:**
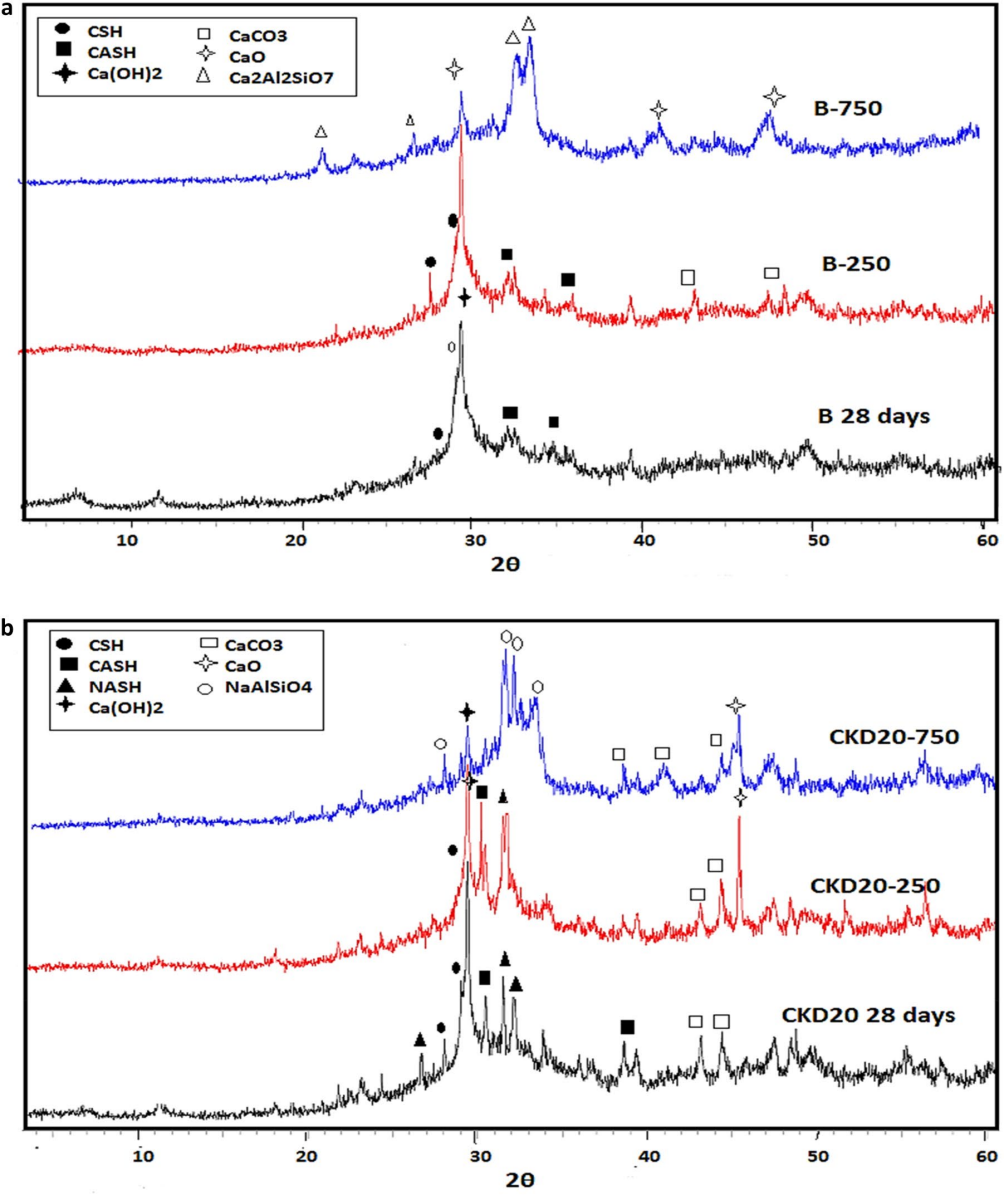
(**a**) XRD patterns of B after firing at 250 and 750 °C. (**b**) XRD patterns of CKD20 after firing at 250 and 750 °C.

**Figure 11:**
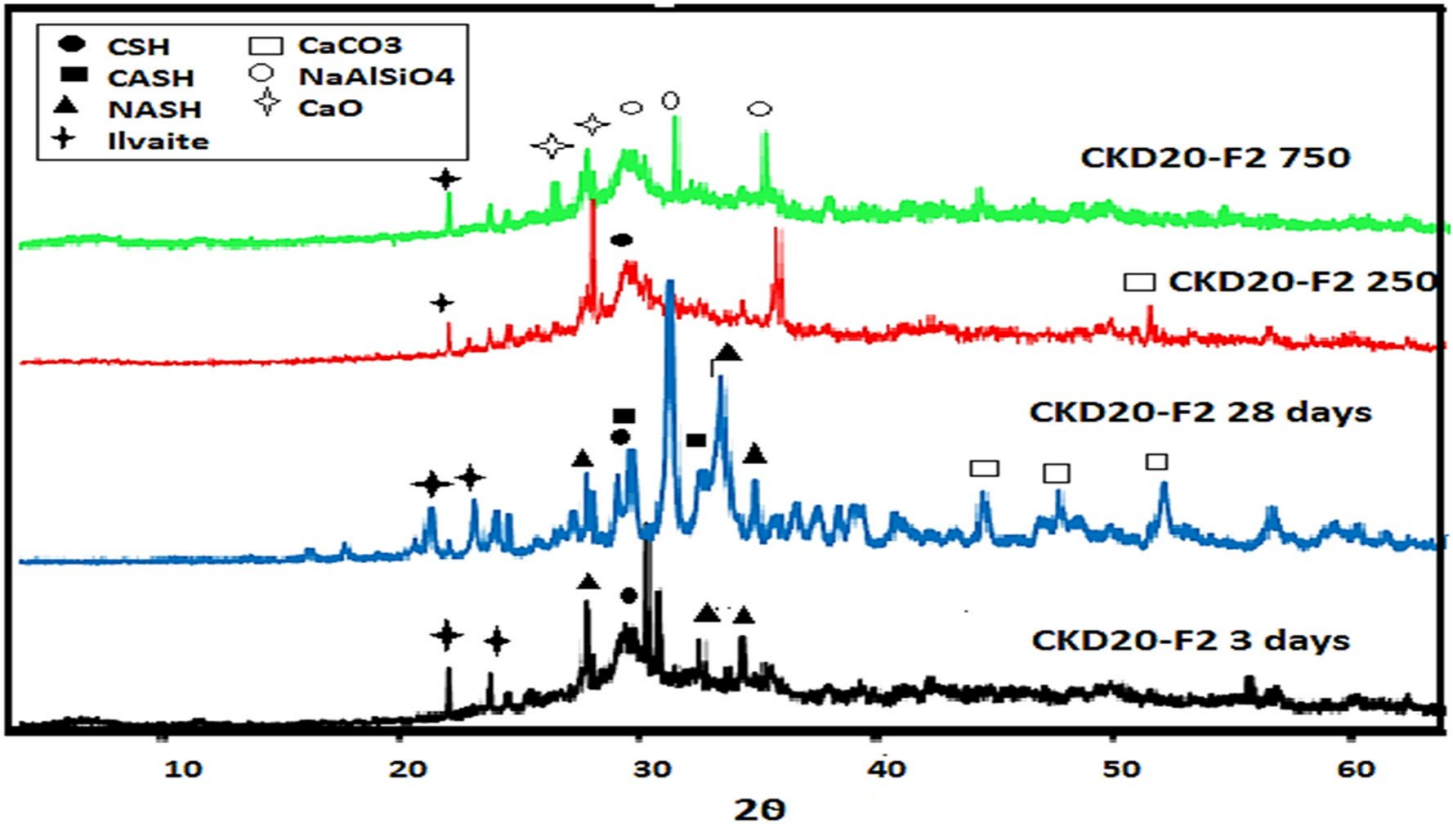
XRD patterns of CKD20 after 3 and 28 days of curing and firing at 250° & 750C.

After 28 days, the peak intensities of the hydrated phases for B and CKD20 in XRD patterns were increased assigned the continuity of the polymerization reactions. XRD patterns of B and CKD20 after firing at 250 and 750°°C were displayed in Fig. 10a,b. XRD pattern of B mix at 250 °C with increasing the sharpness of the peaks related to CSH gel and (CASH) due to the hydrothermal reactions happened by firing at 250 °C^71^. Firing at 750 °C results in nearly disappearance of the hydrated phases as indicting in XRD pattern and formation of dehydrated phases like gehlenite (Ca_2_Al_2_SiO_7_) which has low mechanical properties. XRD patterns of CKD20 at 250 °C shows a similar observation with a decrease in the intensity of peaks related to NASH. At 750 °C, new peaks are detected in XRD pattern which can be related to thermally stable sodium rich phases like nepheline^76^. XRD pattern of CKD20-F2, Fig. 11 shows peaks for CSH, CASH with relative high intensities relative to the those obtained in case of control mix or CKD20 mix. Besides, peaks of Ilavite phase (FCSH) were identified for this composite after 3 and 28 days of curing. This matching the compression strength of CKD20-F2 mix which offered high strength compared to its analogue B and CKD20 mixes. Furthermore, firing CKD20-F2 at 250 °C results in decrease the intensity of peaks related to CSH gel and CASH but still of higher intensities compared to those found in B and CKD20. At 750 °C, nearly the peaks of hydrated phases disappeared due to their decomposition. While peaks due to NaAlSiO_4_ were identified.

### SEM

**Figure 12:**
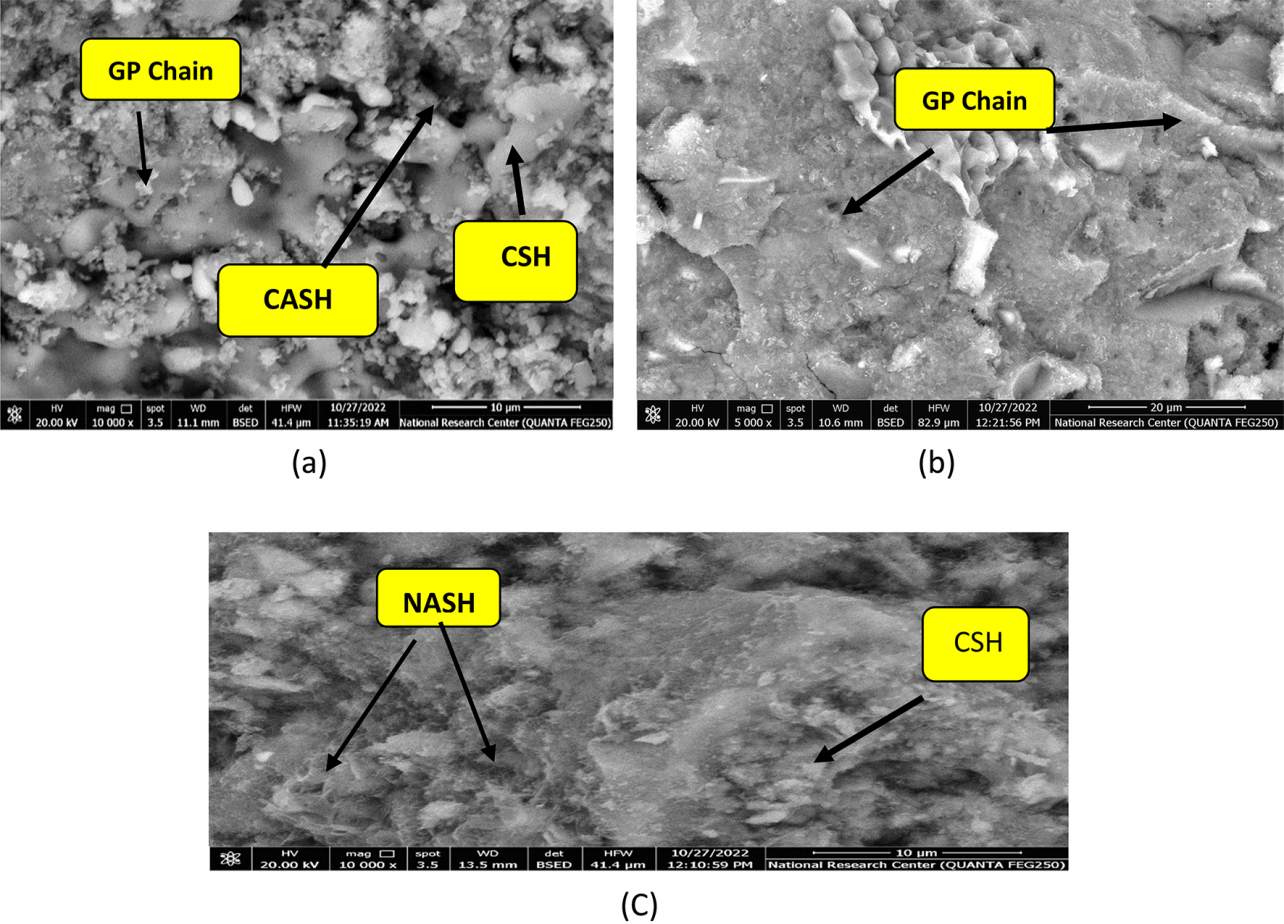
SEM micrographs of GP mixes after 28 days of curing (**a**) B, (**b**) CKD20, (**c**) CKD20-F2.

**Figure 13:**
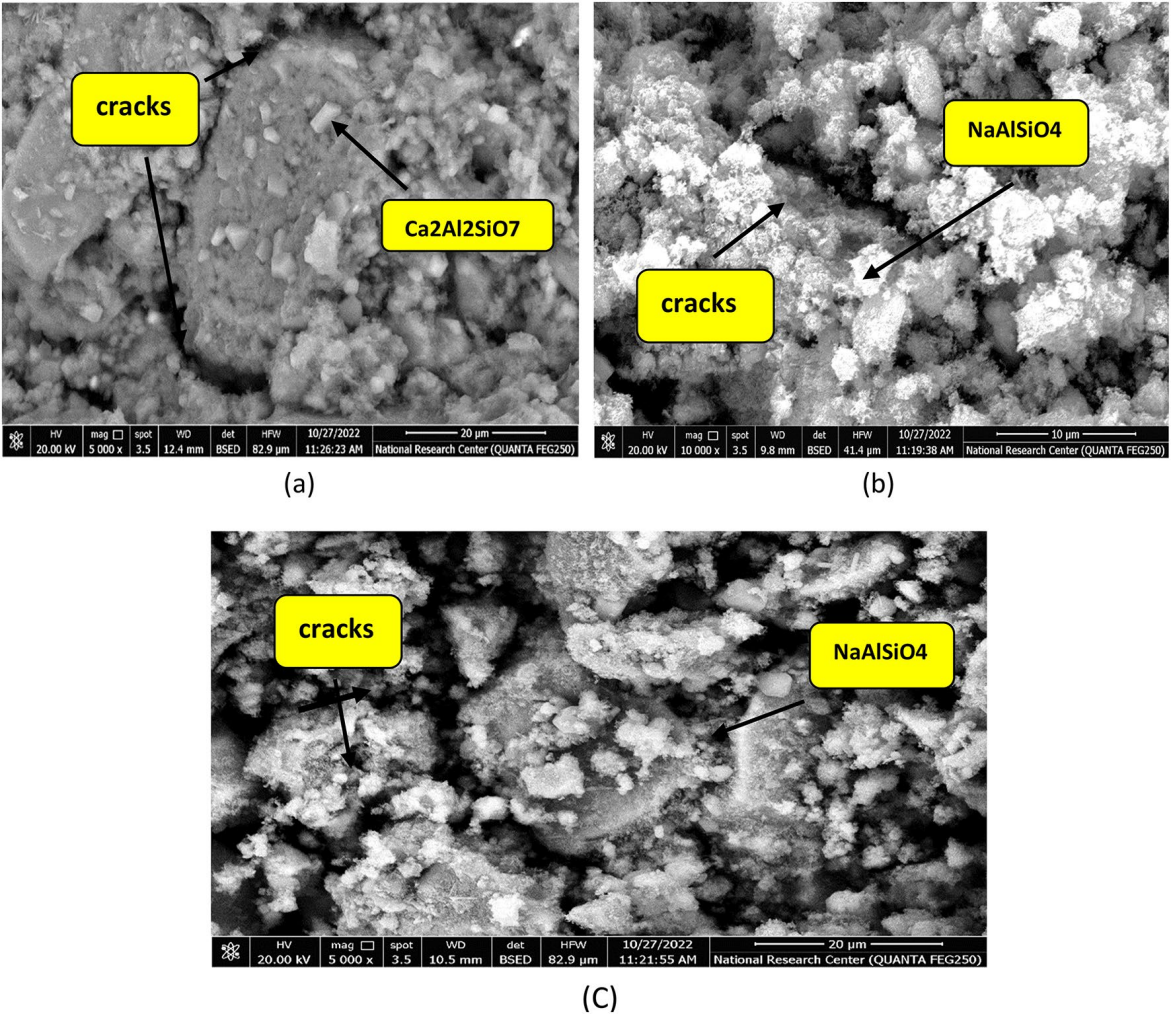
SEM micrographs of GP mixes after firing at 750° C (**a**) B, (**b**) CKD20, (**c**) CKD20-F2.

**Figure 14:**
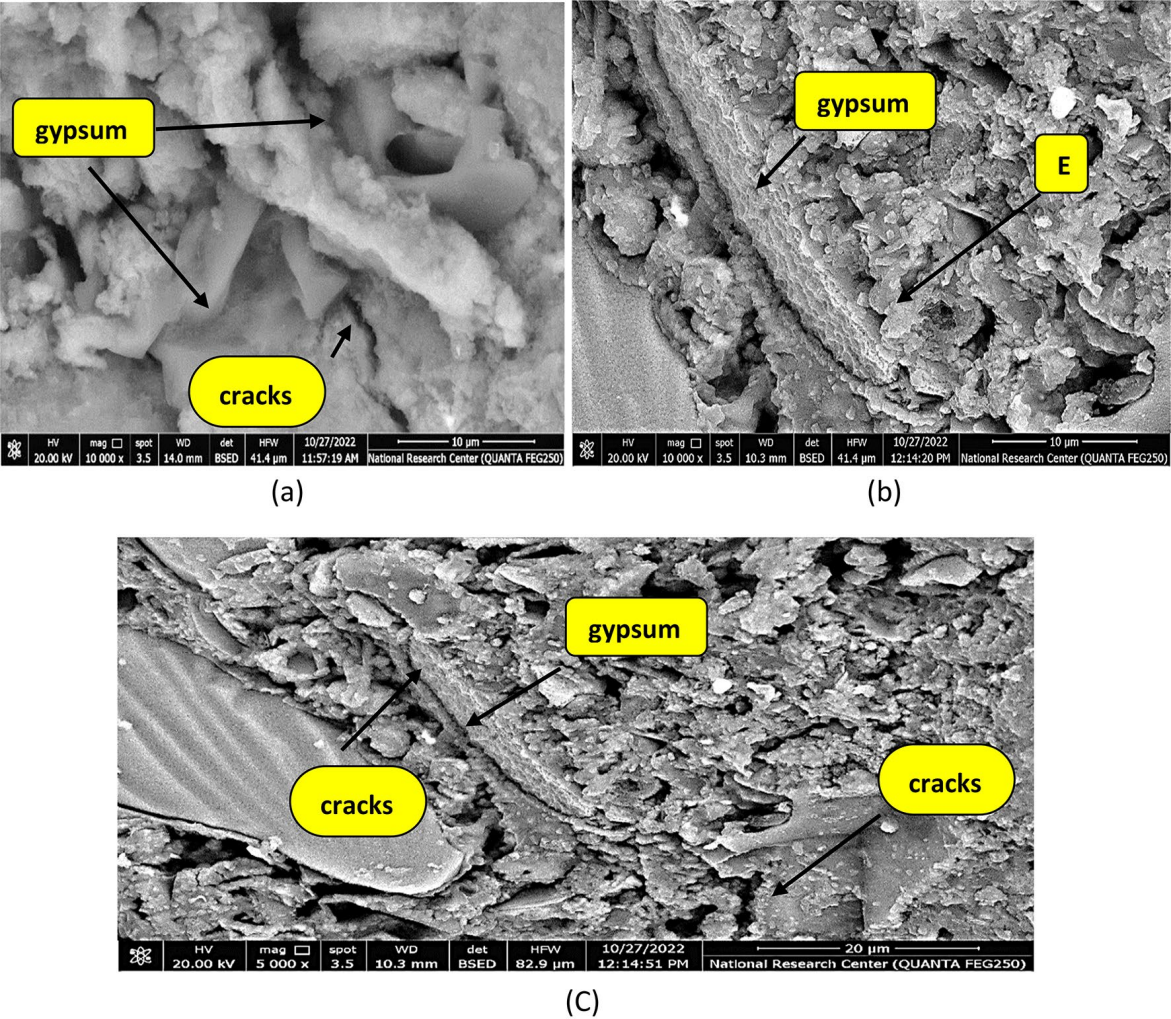
SEM micrographs of GP mixes after attack by sulfuric acid (**a**) B, (**b**) CKD20, (**c**) CKD20-F2.

Microstructure of B, CKD20 and CKD20-F2 after 28 days is studied by SEM micrographs and the images are illustrated in Fig. 12. SEM image of control mix displays a compact structure in which the unreacted spherical slag particles are partially covered by CSH gel and cubic plates of CASH^77^. CKD20 image showed symmetrical and organized clusters of CSHs besides small needles-like crystals are assigned for NASH. In CKD20-F2 image, more gel of CSH is formed besides NASH crystals. SEM images of B, CKD20 and CKD20-F2 samples after firing at 750 °C for 3 h show formation of microcracks beside crystals of unhydrous phases like cubic gehlenite (Ca_2_Al_2_SiO_7_) in B image or plates of nepheline (NaAlSiO_4_) in CKD20 and CKD20-F2 mixes, Fig. 13a–c. These crystalline phases possess low mechanical properties with low interconnecting which explain the failure in CS in these fired samples^78^. Exposure GP samples to attack by H_2_SO_4_ for 60 days clearly affected the GP hydration products as appeared in SEM micrographs, Fig. 14a–c. Gypsum was precipitated on GP chains in these images with appearance of Ettringite needles (E)^79^. Precipitation of these expansive products (E and Gypsum) induces internal pores pressures leading to cracks formation. This explain why the cracks were shown in all mixes which explain the dramatic failure in CS and the less resistivity of the fabricated GP to action of sulfuric acid^62,65,80^.

## Conclusions

In this study, we prepared a one-mix slag/CKD-based geopolymer reinforced with NF or NT (0.5, 1%) to act as sustainable cementitious composite can replace the traditional of high PC in many applications. From the obtained results, we can conclude the following:

1. Slag/CKD-based geopolymers offered longer setting times and higher water demand than did the control (100% slag).
2. Slag/CKD modified by NPs causes a decrease in the setting times to become matching the control mix, and NF results in faster setting than does NT.
3. Including up to 60% of the slag in the CKD treatment reduced the compressive strength. This was related to the change in the GP oxide composition upon blending, and the percentage of Si and Al decreased with increasing CaO content. However this decline in strength in slag/CKD could be compensate by reinforcing the mixes either by 1% NT or NF.
4. All slag/CKD-based geopolymers (free and reinforced with NPs) exhibited a low resistance to attack by H_2_SO_4,_ and ~ 50% of the compressive strength in all the mixes was lost by immersion in H_2_SO_4_ solution for 60 days.
5. The composite mixture offered promising resistance to attack by SO_4_^2–^ ions. CKD20 retained approximately 80% of its strength after immersion in MgSO_4_ solution (7%) for 4 months, while CKD20-F2 and CKD20-T2 retained ~ 91 and 88%, respectively, of their strength during the same period.
6. Slag/CKD-based GPs and reinforcement by NPs showed better firing performance and could retain 80–102% of their strength by firing at 500 °C and 50–55% at 750 °C.
7. In addition, they resisted the destructive effect of γ-ray irradiation up to 1500 *kGy* and retained ~ 75% of their strength after irradiation at dose 1500 kGy, while the strength of the control mixture was only 35%.
8. The fabricated composites are recommended for secure use as lining of medical and scientific labs, mortar applications, restoration supplies, and pavement construction

## Data Availability

The datasets used and/or analyzed during the current study available from the corresponding author on reasonable request.
